# Limited Transplantation of Antigen-Expressing Hematopoietic Stem Cells Induces Long-Lasting Cytotoxic T Cell Responses

**DOI:** 10.1371/journal.pone.0016897

**Published:** 2011-02-17

**Authors:** Warren L. Denning, Jun Xu, Siqi Guo, Christopher A. Klug, Zdenek Hel

**Affiliations:** 1 Department of Microbiology, University of Alabama at Birmingham, Birmingham, Alabama, United States of America; 2 Department of Pathology, University of Alabama at Birmingham, Birmingham, Alabama, United States of America; University of Rochester, United States of America

## Abstract

Harnessing the ability of cytotoxic T lymphocytes (CTLs) to recognize and eradicate tumor or pathogen-infected cells is a critical goal of modern immune-based therapies. Although multiple immunization strategies efficiently induce high levels of antigen-specific CTLs, the initial increase is typically followed by a rapid contraction phase resulting in a sharp decline in the frequency of functional CTLs. We describe a novel approach to immunotherapy based on a transplantation of low numbers of antigen-expressing hematopoietic stem cells (HSCs) following nonmyeloablative or partially myeloablative conditioning. Continuous antigen presentation by a limited number of differentiated transgenic hematopoietic cells results in an induction and prolonged maintenance of fully functional effector T cell responses in a mouse model. Recipient animals display high levels of antigen-specific CTLs four months following transplantation in contrast to dendritic cell-immunized animals in which the response typically declines at 4–6 weeks post-immunization. Majority of HSC-induced antigen-specific CD8^+^ T cells display central memory phenotype, efficiently kill target cells in vivo, and protect recipients against tumor growth in a preventive setting. Furthermore, we confirm previously published observation that high level engraftment of antigen-expressing HSCs following myeloablative conditioning results in tolerance and an absence of specific cytotoxic activity in vivo. In conclusion, the data presented here supports potential application of immunization by limited transplantation of antigen-expressing HSCs for the prevention and treatment of cancer and therapeutic immunization of chronic infectious diseases such as HIV-1/AIDS.

## Introduction

CTLs play a key role in the immune-mediated control of cancer and various infectious diseases. However, endogenous antigen-specific effector T cells induced by transformed cells or invading pathogens are continually depleted by processes of functional exhaustion, anergy, and activation-induced cell death [1]–[5]. Multiple clinical trials have demonstrated that the administration of autologous dendritic cells (DCs) presenting tumor-specific antigens represents a highly effective method of eliciting tumor-specific CTLs. However, this strategy appears to have limited therapeutic effect due to the limited longevity and migratory capacity of injected DCs [6]–[10]. Similarly, adoptive transfer of ex vivo-expanded autologous tumor antigen-specific CTLs for cancer immunotherapy is restricted by the fact that expanded T cells display limited survival upon transfer to recipients and long-lasting remissions are observed only in a fraction of patients [1], [11]. In chronic viral infections such as human immunodeficiency virus-1 (HIV-1) infections in humans or simian immunodeficiency virus (SIV) infection in macaques, we and others have shown that therapeutic immunization of infected animals with recombinant viral vaccines inducing cellular responses improves their ability to control infection in the absence of anti-retroviral therapy; however, vaccine-induced antigen-specific CTLs rapidly decline to pre-immunization levels within weeks post immunization [3], [12]–[14]. The transient nature of vaccine-induced immune responses represents an important problem in the fields of vaccinology, immunotherapy of cancer, and immunotherapy of HIV-1/AIDS. New strategies improving long-term maintenance of functional CTLs are critically needed for an efficient immunotherapy of cancer and chronic infectious diseases.

The potential of genetically modified HSCs to sustain multilineage reconstitution in autologous or heterologous hosts resulting in corrections of various diseases has been well demonstrated [15]–[19]. The pluripotent nature of HSCs allows them to differentiate into multiple cell lineages including professional antigen presenting cells (APCs). Mobilized human CD34^+^ HSCs can be isolated in large numbers and genetically modified by transduction with lentiviral vectors encoding the genes of interest under a control of a target lineage-specific promoter. Importantly, recent advances in the design of self-inactivating lentiviral vectors resulted in a significant increase in the safety of lentivirally-modified HSCs and spurred a number of newly initiated clinical trials [17]–[19]. Several previously published reports have addressed potential application of immunization with genetically modified HSCs [20]–[22]. However, measurable immune responses and control of tumor growth were achieved only by adopting complex protocols involving adoptive transfer of antigen-specific transgenic T cells and co-administration of anti-CD40, Flt3, GMCSF and/or CpG.

In this report we investigate a novel approach to long-term elicitation of CD8^+^ T cells in vivo based on a limited transplantation of genetically modified autologous HSCs resulting in a continually renewing reservoir of cells presenting and/or cross-presenting the antigen. This would result in a reduction of the number of administrations of cellular vaccines needed for long-term maintenance of antigen-specific responses. We demonstrate that induction of a limited antigenic microchimerism by transplantation of low numbers of genetically modified HSCs in combination with nonmyeloablative conditioning results in an expansion of antigen-specific T cells displaying central memory phenotype and providing prolonged protective CTL responses. In contrast, high-level transplantation of antigen-expressing HSCs into fully myeloablated recipients results in an induction of T cell anergy and absence of antigen-specific target cell killing in vivo.

## Materials and Methods

### Ethics Statement

All experimental procedures were approved by the Institutional Animal Care and Use Committee at the University of Alabama at Birmingham (approval number 071207243 and 070507485).

### Mice

C57Bl/6 (B6) and SJL mice were obtained from Harlan Laboratories (Indianapolis, IN); OVA-transgenic mice expressing chicken OVA under the control of chicken β-actin promoter/CMV immediate-early enhancer were obtained from Jackson Labs (Bar Harbor, ME; strain # 005145).

### Isolation and transplantation of HSCs

Bone marrow cells were harvested from the radius, tibias, femurs, and humerus of donor animals and resuspended in complete RPMI medium (RPMI-1640, 0.3 mg/ml L-glutamine, 100 U/ml penicillin, 100 U/ml streptomycin, 50 µM 2-mercaptoethanol [Invitrogen, Carlsbad, CA] supplemented with 10% heat-inactivated fetal bovine serum [FBS, Cellgro, Mediatech Inc, Manassas, VA]). Bone marrow cells were filtered through a 70 µm Nylon mesh (Falcon, Franklin Lakes, NJ) and erythrocytes were lysed in RBC Lysis Buffer (Biolegend, San Diego, CA). Lineage negative (Lin^−^) cells were enriched from bone marrow cells using the Lineage Cell Depletion kit (Miltenyi Biotec, Auburn, CA) and stained with anti-Sca-1 and c-kit antibodies (BD Biosciences, San Jose, CA). Lin^−^ Sca-1^+^ c-kit^+^ (LSK) cell population was sorted on a FACSVantage (BD Bioscience). Typically, 6-7×10^4^ LSK cells were isolated from each 6–8 week old female B6 mouse.

Two days prior to transplantation, mice were pretreated with 60 mg/kg busulfan (BU; Sigma, St. Louis, MO) in 300 µl PBS administered I.P. Nonmyeloablative dose of BU selectively kills HSCs thus making stem-cell niches available for the engraftment of donor HSCs [23], [24]. Alternatively, a single dose of radiation was administered at the indicated amount on the day of transplantation. In experiments involving administration of 900 RADs, the total time of exposure was split into two with a 10 minute rest period in between. Irradiated mice received 1.1 g/L of neomycin sulfate (Mediatech Inc., Manassas, VA) in drinking water two days prior to irradiation and for two weeks post-irradiation. 500 - 2×10^4^ cells were injected intravenously (i.v.) into the lateral tail vein of the recipient animal.

### DC Immunization

Bone marrow-derived dendritic cells were prepared from harvested bone marrow cells by 8 day incubation in the presence of 20 ng/ml GM-CSF (R&D systems, Minneapolis, MN) as previously described [25]. Mice were immunized by i.v. or foot pad injection with indicated numbers of DCs derived from OVA-transgenic mice (DC-tOVA) or DCs from B6 mice coated for 2 hrs with 10 µM of OVA-1 and OVA-2 peptides in complete RPMI medium (DC-pOVA). MHC class I-restricted OVA-1 (OVA_257–264_, SIINFEKL) and MHC class II-restricted OVA-2 (OVA_323–339_, ISQAVHAAHAEINEAGR) peptides were synthesized by Genemed Synthesis (South Francisco, CA).

To assess the relative contribution of various hematopoietic lineages to the induction of antigen-specific CD8^+^ T cells in HSC-immunized recipients, individual cell populations were purified from OVA-transgenic mice. B cells and DCs were sequentially purified from homogenized spleen and lymph node cells using CD19^+^ and CD11c^+^ MicroBeads kits and MACS columns (Miltenyi Biotec). The CD19^−^CD11c^−^ cell fraction was then stained with anti-CD3, CD49 and CD11b antibodies (BD Biosciences) and sorted into T cell (CD3^+^), NK cell (CD3^−^CD49^+^), macrophage/monocytes (CD3^−^CD49^−^CD11b^+^), and other (CD3^−^ CD49^−^ CD11b^−^) cell populations using FACSAria cell sorting system (BD). Cell populations were collected in complete RPMI 1640, washed, and resuspended in RPMI1640 without additives. 2×10^4^ cells of each population were injected i.v. into the tail vein of recipient animals.

### Pentamer and cell surface marker staining

Following erythrocyte lysis, splenocytes homogenized into a single cell suspension or peripheral blood mononuclear cells (PBMCs) were incubated with PE-conjugated OVA-1-specific MHC-I pentamer (ProImmune, Springfiled, VA) for 40 min at 4°C. Cells were washed and stained with anti-CD8 (BD), CD62L, CD27, or CD127 (eBiosciences, San Diego, CA) antibodies for additional 20 min at 4°C prior to washing and flow cytometry analysis (FACSCalibur, BD). In experiments where SJL (CD45.1^+^) and B6 (CD45.2^+^) were used as donors and recipients, respectively, the level of engraftment was determined by collecting 100 µl of blood from the tail vein and staining the obtained PBMCs with CD45.1 and CD45.2 antibodies (BD) for 20 min at 4°C.

### *In vivo* cytotoxic T cell assay

10^7^ autologous splenocytes from wild-type B6 mice were labeled using 0.35 µM 5-(and 6)-carboxyfluorescein diacetate succinimidyl ester (CFSE; control population) and 10^7^ autologous splenocytes from OVA-transgenic mice were labeled using 3.5 µM CFSE (target population) according to the manufacturer's protocol (Invitrogen). Both populations were simultaneously administered i.v. into the tail vein. Under these conditions, only splenocytes from OVA-transgenic mice (high intensity peak on the right) will be recognized by recipient's OVA-specific CTLs. Spleens of immunized and control animals were harvested 24 hrs later, homogenized into single cell suspensions, red blood cells were lysed and target cell killing was determined by flow cytometry analysis (FACSCalibur, BD). Relative killing ratio was calculated by dividing CFSE High/Low ratio in individual treated animals by the average ratio observed in untreated control animals; the values subtracted from 100% represent the percentage of target cells killed.

### Tumor protection assay

16 weeks post-HSC injection, mice were inoculated subcutaneously in left flank with 10^6^ thymoma-derived E.G7 cells expressing OVA [26]. Tumor size was monitored every two days by measuring two perpendicular diameters and the tumor volume was calculated using the formula: (width)^2^ × length.

### Statistical Analysis

All reported P values are two-sided. Group comparisons were performed using the Mann-Whitney rank sum test and repeated-measures ANOVA (RM-ANOVA). Correlations were performed using Spearman rank order test. The SigmaStat (SPSS, Chicago, IL) and GraphPad Prism (GraphPad Software Inc., LaJolla, CA) statistical and graphing software packages were used.

## Results

### Transplantation of genetically modified HSCs results in an improved longevity of vaccine-induced antigen-specific CD8^+^ T cell responses

**Figure 1:**
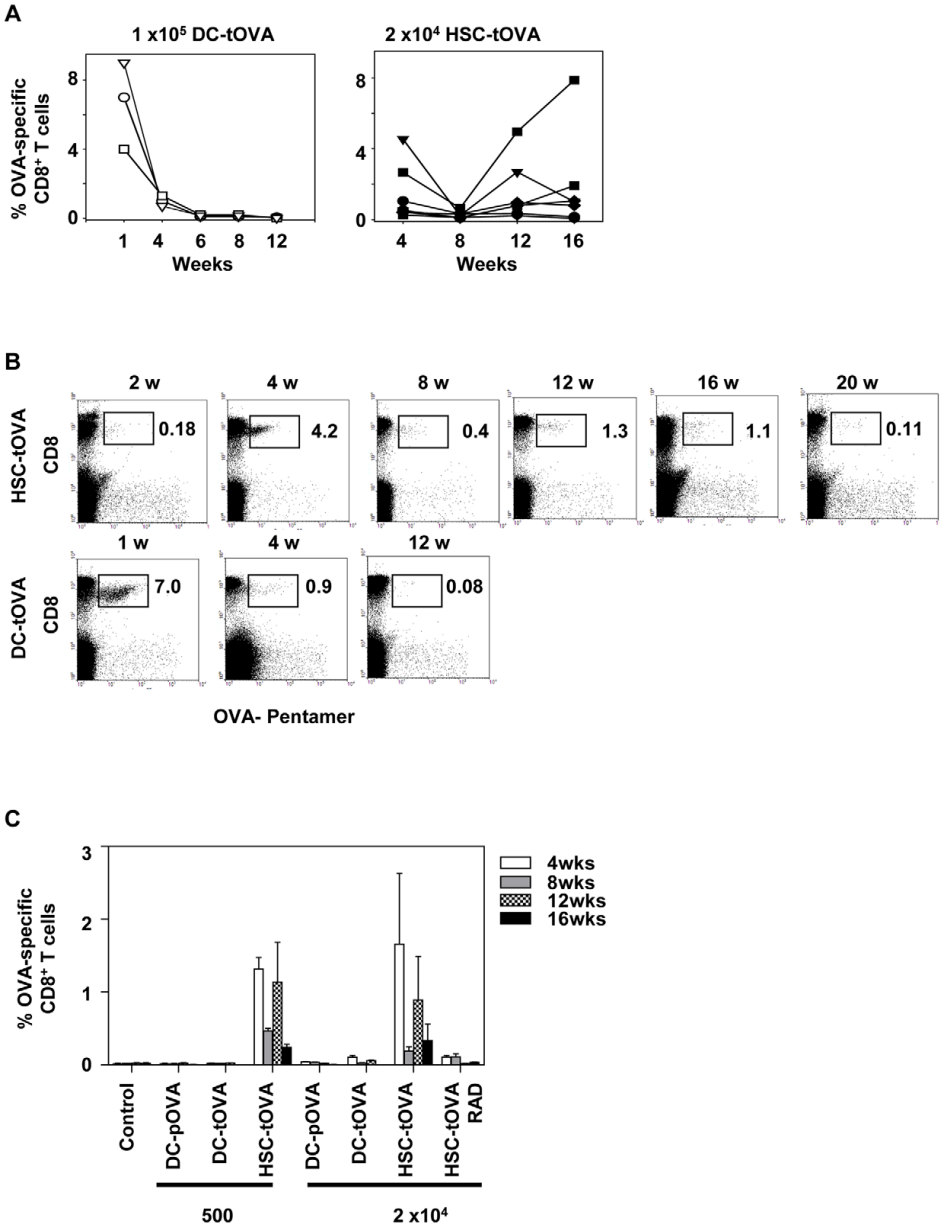
Transplantation of genetically modified HSCs following nonmyeloablative conditioning results in a prolonged maintenance of antigen-specific CD8^+^ T cells. (A) B6 mice were immunized i.v. with 10^5^ DCs from OVA-transgenic mice (DC-tOVA, left panel) or pretreated with BU (60 mg/ml) and two days later transplanted with 2×10^4^ OVA-expressing HSCs (HSC-tOVA, right panel). Percentages of OVA-specific CD8^+^ T cells out of total CD8^+^ leukocytes were determined in blood of immunized animals at the indicated time points by staining with OVA-specific MHC-I pentamer and flow cytometry analysis. (B) Examples of flow cytometry analysis of OVA-specific CD8^+^ T cell immune responses in blood of two representative animals. (C) Mice were immunized with 500 or 2×10^4^ DC-tOVA, immunodominant OVA peptide-coated DCs (DC-pOVA), or HSC-tOVA following BU pretreatment and the percentages of OVA-specific CD8^+^ T cells out of total CD8^+^ cells in blood were determined by OVA-specific MHC-I pentamer staining at indicated time points. Mice in the HSC-tOVA/RAD group were exposed to a split dose of 900 RADs prior to transplantation of 2×10^4^ HSC-tOVA. 4 animals per group; error bars represent standard errors. Representative results of two of five similar experiments are presented.

**Figure 2:**
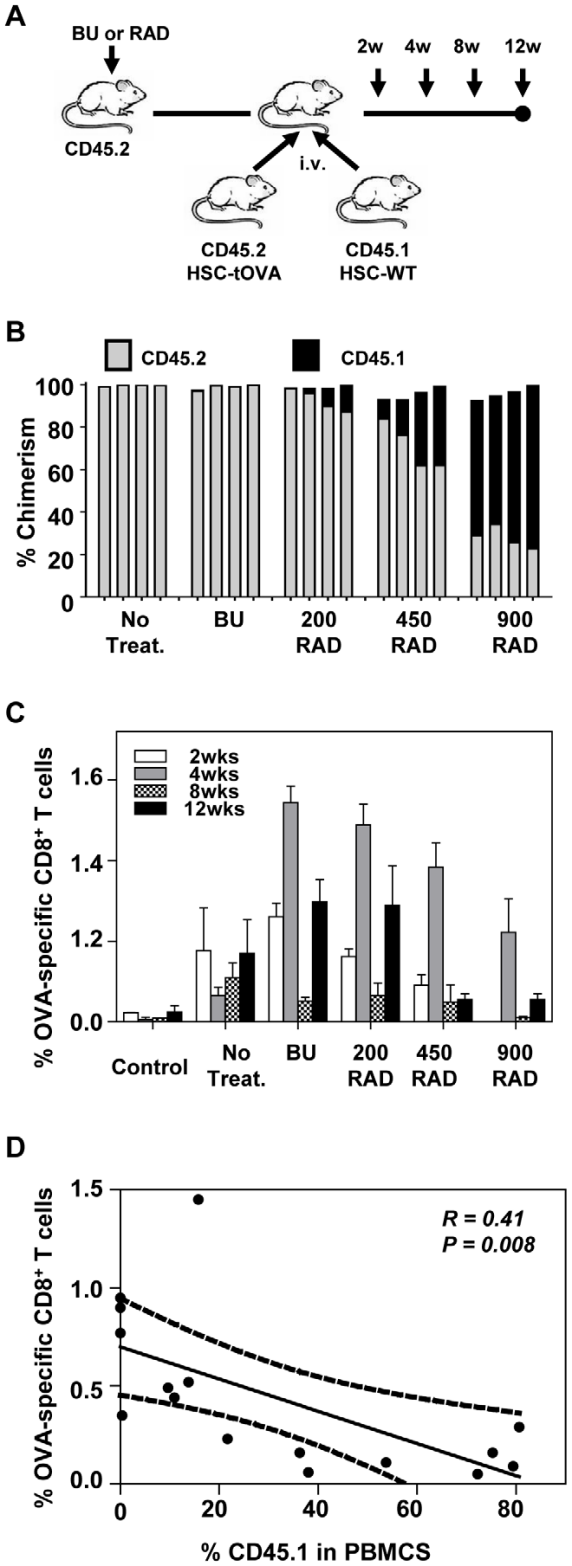
Low level engraftment of transgenic HSCs is required for elicitation of high frequencies of antigen-specific CD8^+^ T cells. (A) Experimental design. Recipient B6 mice (CD45.2^+^) were not treated, treated with BU, or exposed to varying levels of radiation as described in Methods. 10^4^ HSC-tOVA from OVA-transgenic mice on B6 background (CD45.2^+^) and 10^4^ HSCs from SJL mice (CD45.1^+^) were co-administered into the B6 recipients. (B) Percentages of chimerism detected in the blood of recipient animals at indicated time points (4 mice per group). In some instances total values do not amount to 100% due to the presence of cells falling into the CD45.1^−^CD45.2^−^ and CD45.1^+^CD45.2^+^ populations. (C) Percentages of OVA-specific CD8^+^ T cells in blood determined by OVA-specific MHC-I pentamer staining at indicated time points. (D) Inverse correlation between the level of engraftment and induction of antigen-specific CD8^+^ T cells (week 12 post HSC transplantation; analyzed by Spearman rank order correlation test).

To address whether sustained low-level expression of antigen results in an induction and maintenance of antigen-specific CTLs, recipient C57Bl/6 (B6) mice were transplanted with HSCs from OVA-transgenic donor mice on B6 background (HSC-tOVA). Two days prior transplantation, recipient animals were non-myeloablatively conditioned with busulfan (BU; 60 mg/kg i.v.) [23], [24]. HSC recipients were injected i.v. with a single dose of 500 or 2×10^4^ of HSC-tOVA. Control groups were injected i.v. with 10^5^ DCs derived from OVA-transgenic donors (DC-tOVA). The frequency of OVA-specific CD8^+^ T cells in DC-immunized mice peaked at 1 week after immunization and rapidly declined thereafter (Fig. 1A, left). In contrast, in HSC-tOVA-immunized mice, OVA-specific CD8^+^ T cells appeared with slower kinetics reaching high frequencies at weak 4 post transplantation (Fig. 1A, B). This is consistent with the appearance of circulating donor cells at 3–4 weeks post transplantation as evidenced by the occurrence of chimerism in CD45.2^+^ controls transplanted with CD45.1^+^ Lin^−^ Sca-1^+^ HSCs (Fig. 2B and data not shown) [22]. Considerable variations in the frequencies of antigen-specific CD8^+^ T cells were observed among individual animals (Fig. 1A). A significant decrease in the frequency of antigen-specific T cells consistently occurred at 8 weeks post HSC transplantation followed by an increase at 12 weeks. This phenomenon is consistent with differences in the kinetics of appearance of cells descending from long-term self-renewing pluripotent HSCs versus short-term HSCs and partially differentiated lineage-committed common myeloid and lymphoid progenitors [22], [27]–[30]. The increase in the frequency of antigen-specific cells at 12 weeks suggests continuous low-level antigen expression. High levels of OVA-specific T cells were detectable at 16 weeks post vaccination but declined in most recipients at 20–24 weeks post treatment. Compared to transgenic DCs or DCs coated with a specific immunodominant peptide (DC-pOVA), administration of as few as 500 OVA-expressing HSCs into busulfan-pretreated animals resulted in a maintenance of significantly higher frequencies of OVA-specific cells at 4–16 weeks post immunization (Fig. 1C) (*p*<0.03 for HSC-tOVA versus DC-pOVA and HSC-tOVA versus DC-tOVA comparisons at 500 and 2×10^4^ HSC vaccine doses at weeks 12 and 16). Pre-treatment of differentiated DC-tOVA or DC-pOVA with LPS (bacterial lipopolysaccharide, 100 ng/ml, 24 hrs) or administration of DCs subcutaneously into foot pad did not significantly enhance the longevity of induced immune responses determined as frequency of antigen-specific cells at weeks 4–16 post vaccination ([31] and data not shown). Importantly, mice transplanted following myeloablative conditioning (lethal irradiation, 900 RAD) with 2×10^4^ HSCs displayed significantly lower levels of frequencies of antigen-specific CD8^+^ T cells throughout the observation period (Fig. 1C).

Following transplantation, hematopoietic cells expressing non-self antigen are continually removed by host's cellular responses. To address the relationship between the level of engrafted antigen-expressing cells and resulting antigen-specific CD8^+^ T cell responses, B6 mice (CD45.2^+^) were treated with a single dose of BU or exposed to varying levels of radiation. Two days after BU treatment or at the day of irradiation, respectively, mice were administered with an equal number (10^4^) of HSC-tOVA (B6 background, CD45.2^+^) and normal HSCs from SJL mice (CD45.1^+^) (Fig. 2). Under these conditions, only OVA-expressing hematopoietic cells derived from HSC-tOVA are targeted by host's CTLs while the SJL-derived hematopoietic cells are not recognized by recipient's immune system and can serve to determine the relative level of engraftment. The levels of hematopoietic chimerism and the frequencies of antigen-specific CD8^+^ T cell responses were determined in blood at 2 to 12 weeks post transplantation. While untreated mice and BU-pretreated mice displayed less than 0.2% chimerism, radiation-treated mice displayed gradually increasing levels of chimerism up to 80% in lethally irradiated (900 RAD) mice (Fig. 2B). Increasing level of chimerism inversely correlated with the frequencies of antigen-specific CD8^+^ cells (Fig. 2C and D)(*p* = 0.0002 and 0.008 at weeks 4 and 12, respectively). Optimal maintenance of high levels of antigen-specific T cell responses appears to be achieved under nonmyeloablative conditioning (BU or 200 RAD) resulting in a low level of engraftment (<0.2% of transgenic cells of total PBMCs).

In a series of analogous experiments, HSC-tOVA (CD45.2^+^) were transplanted into SJL (CD45.1^+^) mice following various pre-conditioning methods. No CD45.2^+^ leukocytes were detectable in recipients by flow cytometry above the background of the assay (approximately 0.02% of total leukocytes). Similarly, OVA-transgenic mice were crossed to SJL background and resulting HSC-tOVA expressing CD45.1^+^ were transplanted into B6 (CD45.2^+^) recipients. No OVA-expressing CD45.1^+^ leukocytes were detectable in recipient animals by flow cytometry analysis (data not shown). These experiments strongly suggest that antigen-expressing cells are rapidly depleted by recipient's immune system.

### CD8^+^ T cells maintained in recipients of transgenic HSCs develop central memory T cell phenotype

**Figure 3:**
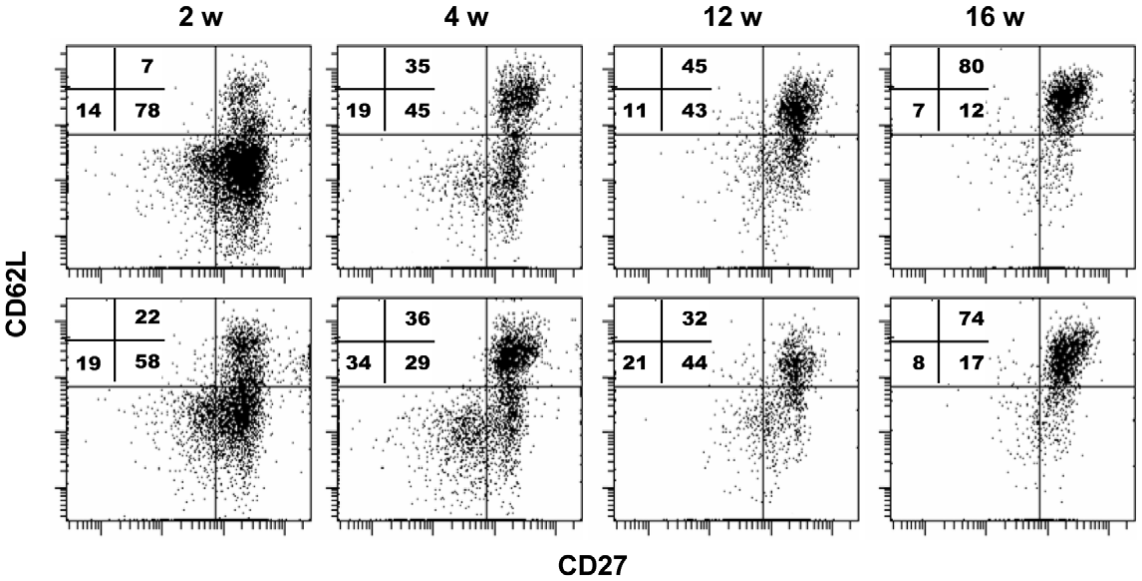
Antigen-specific CD8^+^ T cells maintained in HSC-immunized recipients predominantly display central memory phenotype. 2×10^4^ HSC-tOVA cells were transplanted into BU-conditioned B6 mice. OVA-specific CD8^+^ T cells were detected in splenocytes of immunized mice by staining with OVA-specific pentamer and anti-CD8 antibody. Percentages of antigen-specific CD8^+^ T cells displaying central memory (CD62L^+^ CD27^+^) effector memory (CD62L^−^ CD27^+^), or effector T cell (CD62L^−^ CD27^−^) phenotypes were determined in the spleens of recipient animals at 2, 4, 12, and 16 weeks post transplantation. Data obtained in two animals per time point are presented.

While functional effector memory T cells mediate clearance of tumor and infected cells *in vivo*, central memory T cells appear to play an indispensible role in the long-term control [1], [32]. To investigate the phenotype of CD8^+^ T cells elicited by HSC-tOVA immunization, splenic OVA-specific CD8^+^ T cells were analyzed at 2, 4, 12, and 16 weeks following transplantation of 2×10^4^ HSC-tOVA into BU-pretreated animals. At 2 to 4 weeks post transplantation, antigen-specific CD8^+^ T cells were distributed between central memory (CD62L^+^ CD27^+^) effector memory (CD62L^−^ CD27^+^), and, to a lesser degree, effector (CD62L^−^ CD27^−^) T cell populations (Fig. 3). At 16 weeks, more than 70% of OVA-specific CD8^+^ T cells displayed central memory phenotype. Low production of IL-2, IFN-γ, and granzyme B by OVA-specific memory CD8^+^ T cells harvested at 12 weeks post immunization was observed by intracellular staining assay following 6 hrs of ex vivo stimulation with specific OVA-1 peptide (data not shown). This observation may be related to the constant antigen stimulation of antigen-specific T cells in vivo.

### Antigen-specific T cells induced by immunization with HSC-tOVA efficiently kill target cells *in vivo*

**Figure 4:**
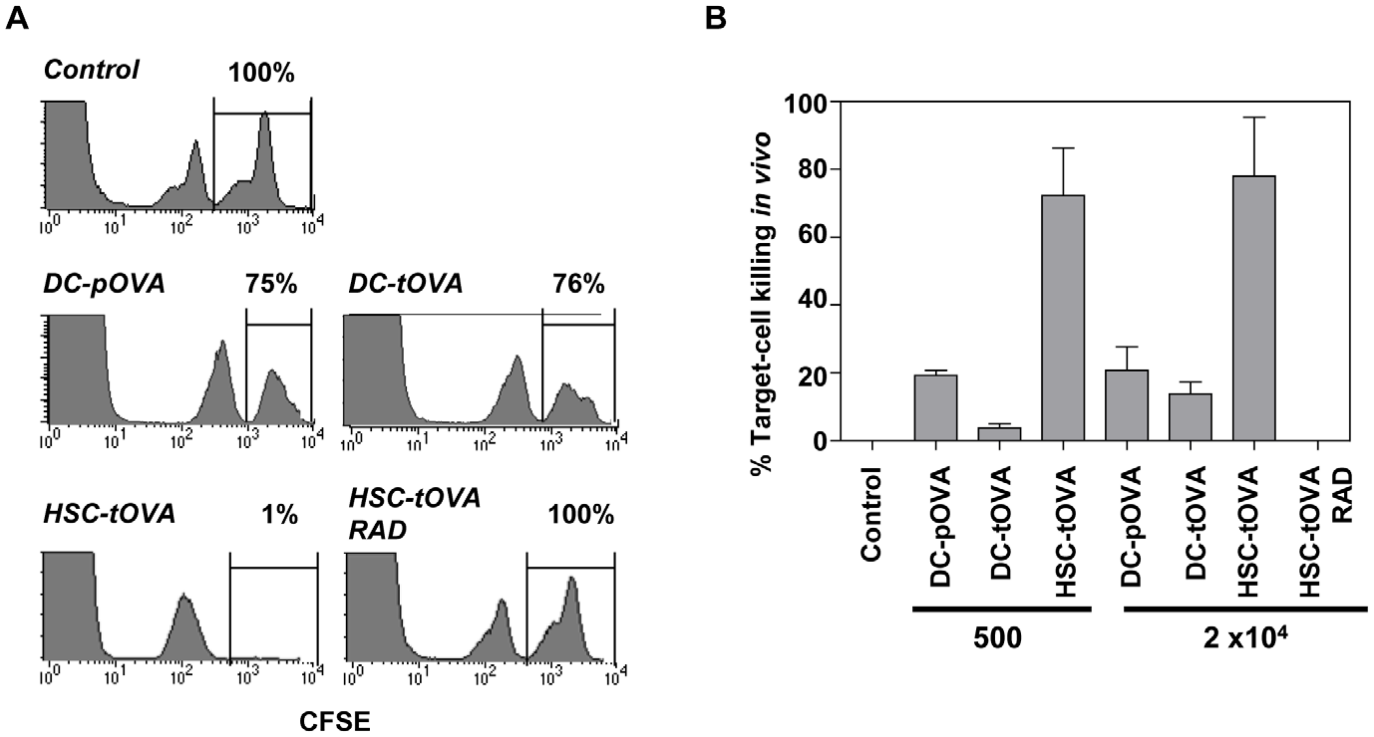
Antigen-specific T cells induced by HSC immunization efficiently kill target cells *in vivo*. (A) B6 mice were immunized with 2×10^4^ DC-tOVA, OVA peptide-coated DCs (DC-pOVA), or HSC-tOVA. HSC-tOVA recipients were pretreated with BU or irradiated with 900 RAD. 16 weeks post immunization, mice were injected with 10^7^ OVA-transgenic splenocytes labeled with high concentration of CFSE (3.5 µM, right peak) and 10^7^ wild type B6 splenocytes labeled with low concentration of CFSE (0.35 µM, middle peak). Under these conditions, only the splenocytes from OVA-transgenic mice (right peak) are target of host OVA-specific CTLs. 24 hours post injection, splenocytes were harvested and the relative proportions of remaining CFSE^+^ populations were determined. (B) Graphical representation of the percentage of target cell killing in vivo. 4 animals per group; error bars represent standard errors.

To test the functionality of antigen-specific T cells induced by administration of HSC-tOVA, the animals were immunized as indicated in the legend of Figure 4. 16 weeks post immunization, immunized animals and controls were administered with 10^7^ CFSE-labeled splenocytes expressing OVA (Fig. 4A, high intensity peak on the right). As internal control, 10^7^ splenocytes from normal B6 mice labeled with low concentration of CFSE were co-injected (Fig. 4A, middle peak). Under these conditions, only splenocytes from OVA-transgenic mice (right peak) will be recognized by recipient's OVA-specific CTLs. 24 hours after injection, splenocytes were harvested and the number of remaining CFSE^+^ cells was determined. The groups administered with 500 or 2×10^4^ HSC-tOVA under nonmyeloablative conditions exhibited significantly greater extent of in vivo target cell killing than mice immunized with OVA-transgenic (DC-tOVA) or OVA peptide-coated DCs (Fig. 4B) (DC-pOVA) (*p*<0.05 for HSC-tOVA/DC-pOVA and HSC-tOVA/DC-tOVA comparisons at both 500 and 2×10^4^ HSC vaccine doses). In contrast, mice that received high dose of radiation (900 RAD) prior to the transplantation of HSC-tOVA did not exhibit any detectable CTL activity. Thus, high-level expression of antigen not only decreases the frequency of antigen-specific CD8^+^ T cells but also abrogates antigen-specific cytotoxic activity.

### Immunization with transgene-expressing HSCs results in a protection against a model tumor

**Figure 5:**
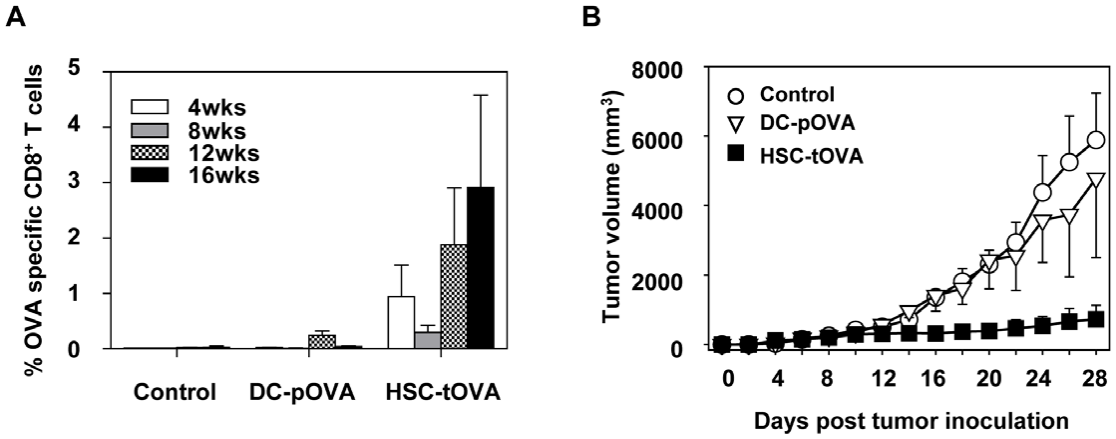
Immunization with genetically modified HSCs results in protection against a growth of a model thymoma. Mice were immunized with 2×10^4^ HSC-tOVA cells following BU pre-treatment or 2×10^4^ immunodominant OVA peptide-coated DCs. (A) Percentages of OVA-specific CD8^+^ T cells in blood were monitored by OVA-specific MHC-I pentamer staining. (B) 16 weeks post immunization, mice were inoculated s.c. with 10^6^ E.G7 thymoma cells expressing OVA. Tumor growth was monitored until mice became moribund. 4 animals per group; error bars represent standard errors.

To test the ability of HSC-based immunization to protect against tumor growth, mice were immunized with 2×10^4^ HSC-tOVA or DCs presenting dominant MHC-I-restricted OVA peptide and 16 weeks later challenged with 10^6^ E.G7 thymoma tumor cells expressing OVA antigen administered subcutaneously. While antigen-specific CD8^+^ T cells were maintained only at low frequencies in DC-immunized animals, immunization with HSCs resulted in an efficient maintenance of specific CTLs up to the time of tumor challenge at week 16 (Fig. 5A). Mice immunized with OVA-transgenic HSCs were able to control tumor growth throughout the observation period significantly better than DC-immunized or control group (Fig. 5B) (*p*<0.05 and *p*<0.001 between HSC-tOVA and DC-pOVA and HSC-tOVA and control group, respectively, weeks 24–28; analyzed by RM-ANOVA). At week 28, two of four HSC-OVA immunized mice but only one of four DC-pOVA-immunized and none of four control mice were free of palpable tumor.

### Multiple hematopoietic lineages may contribute to the induction of antigen-specific CD8^+^ T cells in HSC-immunized animals

**Figure 6:**
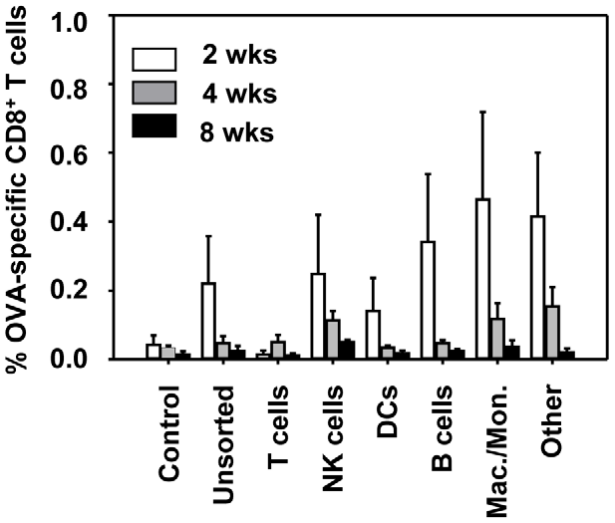
Various hematopoietic lineages likely contribute to the induction of antigen-specific CD8^+^ T cells. Cell populations representing indicated hematopoietic lineages were isolated from the spleen and lymph nodes of OVA-transgenic mice by a combination of magnetic column purification and flow cytometry sorting as described in Methods. B6 mice were immunized i.v. by a single injection of 2×10^4^ cells of the indicated lineage and the percentage of OVA-specific CD8^+^ T cells in blood was determined by pentamer staining at 2, 4, and 8 weeks post immunization. 3 animals per group; error bars represent standard errors.

Specific T cell responses in HSC-OVA recipients can be induced either directly by transgene-expressing professional antigen presenting cells or indirectly by antigen cross-presentation mechanism. To address the mechanism of specific T cell induction, naïve mice were immunized i.v. with 2×10^4^ cells representing individual populations purified from OVA-transgenic mice. Immunization with DCs, B cells, NK cells, and cells of macrophage/monocyte lineage, but not T cells, induced detectable immune responses in recipients at 2 weeks post immunization (Fig. 6). However, in the absence of hematopoietic precursors, immune response subsided by 4 to 8 weeks post immunization.

## Discussion

This study assessed whether administration of antigen-expressing HSCs under nonmyeloablative conditions induces and maintains functional antigen-specific CTL responses for a significantly longer period of time than that typically achieved following immunization with peptide-loaded or antigen-expressing DCs. We demonstrate that transplantation of low numbers of genetically modified HSCs under nonmyeloablative or mildly myeloablative conditions elicits and maintains long-term antigen-specific CD8^+^ T cell responses capable of killing target antigen-expressing cells in vivo. High levels of functional CTLs were readily detectable for 4 months post HSC transplantation and display both effector and central memory phenotypes. HSC-based immunization provided a significant control of the growth of a model tumor in a preventive setting at four months post immunization. To our knowledge, this is the first report demonstrating efficient immunization with antigen-encoding HSCs without a need of a simultaneous transfer of TCR-transgenic T cells and/or complex in vivo stimulation with DC-activating agents such as GM-CSF and CpG. As nonmyeloablative conditioning is increasingly used for the treatment of oncologic disorders [33]–[36], [37], our observations may contribute to the development of novel immunotherapeutic strategies for cancer and therapeutic immunization against some chronic infectious diseases such as HIV-1/AIDS.

Since mobilized peripheral blood-derived CD34^+^ HSCs can be efficiently transduced by lentiviral vectors [38], [39], we believe that genetically modified HSCs can be used as a basis for novel cellular vaccine-based strategies. In contrast to TCR-based strategies, the strategy presented here is independent of the MHC haplotype of the patient; thus, the same lentiviral construct encoding common tumor-associated antigen can be used in all potential recipients. However, the efficacy and safety of HSC-based immunization strategies need to be further evaluated in detailed studies prior to their implementation to clinical practice. Importantly, it remains to be established whether the proposed immunization strategy would efficiently reverse the growth of an established tumor in a therapeutic setting. Results of recently conducted clinical trials employing transplantation of ex vivo-modified HSCs and recombinant lentiviral vectors provide reasons for optimism regarding the safety of this approach [15], [16], [18]. A number of recent clinical trials have demonstrated that HSCs can be modified safely with the current generation of self-inactivating lentiviral vectors and transferred to recipients without elicitation of adverse effects [17]–[19]. The ability of genetically modified HSCs to maintain antigen-specific responses may be further enhanced by targeting antigen expression to specific subsets of antigen-presenting cells by utilization of lineage-specific promoters. In the experiments described here, transplanted HSC-tOVA cells express the alloantigen and therefore are likely to be targeted by recipient's CTLs. Restricting antigen expression to differentiated hematopoietic population such as DCs may significantly enhance the half-life of transplanted HSCs and further prolong the immunization effect.

Current strategies for T cell-based cancer immunotherapy have been selected based on their ability to rapidly expand tumor-specific T cells *in vivo*. However, accumulated data suggest that a single expansion of large quantities of T cells is not sufficient to sustain objective anti-tumor responses in cancer patients [1], [6], [7], [40]. Immunization with high doses of antigen in an environment providing strong costimulatory signals, such as following immunization with activated DCs or recombinant viruses, favors rapid proliferation and differentiation of T cells into Teff cells and ultimately T cells displaying an exhausted phenotype [1], [41]. Successful immunotherapy of cancer requires multiple immunizations over prolonged periods of time to maintain a functional effector T cell population [1], [8], [42], [43]. Suboptimal induction of low frequencies of T cells displaying effector phenotype results in a short-term effect and contributes to the limited efficacy of cellular vaccine-based cancer immunotherapy regimens.

Selective generation of central or early effector memory T cells has been shown in both viral and tumor models to confer superior protective and therapeutic immunity compared to Teff cells [32], [44]–[47]. Several recent reports have shown that in chronic viral infections fully differentiated virus-specific T cells need to be continually replaced by newly primed T cells recently generated in thymus [48], [49]. Newly primed T cells are phenotypically distinct from exhausted T cells and display less differentiated phenotype characterized by higher expression of CD62L, CD27, anti-apoptotic protein bcl-2 and cytokines such as TNF-α and IL-2. This process, at least partially dependent on a functional thymus, prevents the decline of antigen-specific T cells and explains the dependence of T cell “memory” on antigen in chronic infections [50]. Successful T cell-based therapies should aim at continuous induction of central memory/effector T cells at sites distant from the immunosuppressive environment of tumor and draining lymph nodes to avoid immune evasion and induction of tolerance. Since the data presented here suggests that HSC-based immunization results in a prolonged preservation of central memory CD8^+^ T cell responses induced systemically, HSC-based immunotherapy appears to be a highly promising novel approach to CTL-based immunotherapy.

Several previously published reports have addressed the potential application of immunization with genetically modified HSCs [20]–[22]. However, measurable immune responses and control of tumor growth were achieved only by an adoptive transfer of antigen-specific transgenic T cells and co-administration of anti-CD40, Flt3, GMCSF and/or CpG. Application of such complex strategies in clinical setting would be associated with a significant risk and low cost effectiveness. It is likely that HSC transplantation into lethally irradiated recipients in the above cited studies resulted in an induction of central and/or peripheral T cell tolerance. In this regard, our data suggest that transplantation of antigen-expressing HSCs under myeloablative conditions allowing transgenic HSCs to populate a large percentage of the recipient's bone marrow results in a low frequency of antigen-specific T cells (Fig. 1, 2). Moreover, elicited T cells are unable to kill target antigen-expressing cells in vivo (Fig. 4). This phenomenon is consistent with previously published studies [51]–[54]. The mechanism of tolerance induction by HSC transplantation into fully myeloablated recipients was addressed by Chan et al. who demonstrated that transplantation of bone marrow genetically modified to express myelin oligodendrocyte glycoprotein (MOG) prevented the induction and progression of experimental autoimmune encephalomyelitis (EAE) and induced long-term remission of established disease [52]. The mechanism involved was identified as clonal deletion with no evidence for induction of antigen-specific regulatory T cells. Similarly, using the OVA model, Dresh et al. have demonstrated that a transplantation of bone marrow cells genetically modified by lentiviral vector to express OVA antigen under a control of a DC-specific promoter induced central tolerance characterized by clonal depletion of antigen-specific CD4^+^ and CD8^+^ T cells and T cell anergy indicated by the inability of antigen-specific T cells to express TNF-α and IFN-γ and kill antigen-presenting target cells in vivo [51].

The appearance of antigen-specific T cells in recipients of transgenic HSCs is delayed compared to animals immunized with transgenic or peptide-coated DCs. This is consistent with the appearance of circulating donor cells at 3–4 weeks post transplantation as evidenced by the occurrence of chimerism in CD45.2^+^ controls transplanted with CD45.1^+^ HSCs (data not shown). This data is further corroborated by the results of Zhao *et al*. who recently demonstrated that reconstitution of mice following the transplantation of Lin^−^ Sca-1^+^ HSCs is delayed compared to bone marrow transplantation and that first donor-derived DCs occur in the spleen of recipient animals at 3 weeks post transplant [22]. A significant decrease in the frequency of antigen-specific T cells was consistently observed at 8 weeks post HSC transplantation followed by a rebound at 12 weeks. The mechanism underlying this phenomenon is unclear. Recently, it has been realized that purified Lin^−^ Sca-1^+^ c-kit^+^ HSC population consist of CD34^+^ short-term reconstituting cells (STRCs) and CD34^−^ self-renewing long-term reconstituting cells (LTRCs) [28]–[30]. While STRCs sustain clones of differentiating cells for only 4–6 weeks, LTRM-derived hematopoietic cells persist for extended periods of time. In light of these results, it is plausible that the kinetics of appearance of specific T cell responses in HSC immunized animals characterized by a peak at 4 weeks, contraction at 8 weeks, and a subsequent increase at 12–16 weeks post transplantation is driven by the differential appearance of hematopoietic cells derived from STRCs versus LTRCs. The reservoir of dormant LTRCs was shown to be activated in response to inflammation, stress, or injury signals [28]–[30]. This could partially explain the high variability of OVA-specific CD8^+^ T cell frequencies observed in HSC-immunized mice at weeks 12–16 post transplantation.

The mechanism of induction and maintenance of specific T cell responses in HSC-OVA recipients is unclear. Specific T cells can be induced either directly by transgene-expressing professional antigen presenting cells or indirectly by antigen cross-presentation mechanism. The data presented on Figure 6 suggests that immune responses in HSC recipient animals may be induced by multiple cell lineages. In contrast to DCs, macrophages, and B cells known as professional antigen-presenting cells, NK cells do not efficiently present antigens to CD8^+^ T cells. This result suggests that cells derived from antigen-expressing HSCs may induce and maintain specific T cells by a combination of direct antigen presentation and cross-presentation mechanisms [55], [56]. Along similar lines, we have recently published a report demonstrating that efficient immunization with antigen-expressing B cells likely depends on the cross-presentation of the antigen [31]. However, the data presented on Figure 6 do not directly prove an involvement of any of the tested populations in specific T cell induction and more detailed studies are warranted to fully understand the mechanism of HSC-based immunization.

Although more than 33 million people are estimated to live with HIV-1 infection, not a single patient has been successfully cured. The only possible exception is a recipient of HSC transplantation resulting in repopulation of CCR5-negative cells resistant to infection with HIV-1 [37]. We have previously demonstrated that therapeutic immunization of SIV-infected rhesus macaques with CTL-inducing recombinant viral vaccines results in a partial control of infection; however, antigen-specific CTLs expanded following immunization rapidly decline to pre-vaccination levels [3], [12], [13]. HSC immunization may be a promising strategy to long-term maintenance of immune responses in therapeutically immunized HIV-1-infected individuals simultaneously treated with other regimens increasing their resistance to HIV-1 infection.

Important safety concerns need to be addressed before HSC-based immunotherapy can be applied to clinical practice. More research is warranted to address the interplay between the level of HSC engraftment and the resulting induction of specific immune responses versus tolerance. However, the data presented here demonstrate the potential advantage of immunization with low numbers of genetically modified HSCs over conventional DC-based immunization and support application of immunization with HSCs for the treatment and prevention of cancer and chronic infectious diseases such as HIV-1/AIDS.
